# Association of Phenotypic Aging Marker with comorbidities, frailty and inflammatory markers in people living with HIV

**DOI:** 10.1186/s12877-022-03720-1

**Published:** 2022-12-31

**Authors:** Win Min Han, Tanakorn Apornpong, Sivaporn Gatechompol, Sasiwimol Ubolyam, Pairoj Chattranukulchai, Lalita Wattanachanya, Sarawut Siwamogsatham, Stephen J. Kerr, Kristine M. Erlandson, Anchalee Avihingsanon

**Affiliations:** 1 The HIV Netherlands Australia Thailand Research Collaboration (HIV-NAT), Thai Red Cross AIDS Research Centre, Bangkok, Thailand; 2 Centre of Excellence in Tuberculosis, Department of Medicine, Faculty of Medicine, Chulalongkorn University, Bangkok, Thailand; 3 Division of Cardiovascular Medicine, Faculty of Medicine, Chulalongkorn University, King Chulalongkorn Memorial Hospital, Bangkok, Thailand; 4 Division of Endocrinology and Metabolism, Department of Medicine, Faculty of Medicine, Chulalongkorn University, Bangkok, Thailand; 5 Excellence Center for Diabetes, Hormone, and Metabolism, King Chulalongkorn Memorial Hospital, Bangkok, Thailand; 6 Division of Ambulatory and Hospital Medicine, Faculty of Medicine, Chulalongkorn University, Bangkok, Thailand; 7 Chula Clinical Research Center, Faculty of Medicine, Chulalongkorn University, Bangkok, Thailand; 8 Kirby Institute, UNSW Sydney, Sydney, Australia; 9 Biostatistics Excellence Centre, Faculty of Medicine, Chulalongkorn University, Bangkok, Thailand; 10 Division of Infectious Diseases, School of Medicine, University of Colorado-Anschutz Medical Campus, Aurora, CO USA

**Keywords:** Phenotypic aging marker, Phenotypic age acceleration, HIV/AIDS, Frailty, Aging, Comorbidities, Thailand

## Abstract

**Background:**

Aging characteristics in people living with HIV (PLWH) are heterogeneous, and the identification of risk factors associated with aging-related comorbidities such as neurocognitive impairment (NCI) and frailty is important. We evaluated predictors of novel aging markers, phenotypic age (PhenoAge) and phenotypic age acceleration (PAA) and their association with comorbidities, frailty, and NCI.

**Methods:**

In a cohort of PLWH and age- and sex-matched HIV-negative controls, we calculated PhenoAge using chronological age and 9 biomarkers from complete blood counts, inflammatory, metabolic-, liver- and kidney-related parameters. PAA was calculated as the difference between chronological age and PhenoAge. Multivariate logistic regression models were used to identify the factors associated with higher (>median) PAA. Area under the receiver operating characteristics curve (AUROC) was used to assess model discrimination for frailty.

**Results:**

Among 333 PLWH and 102 HIV-negative controls (38% female), the median phenotypic age (49.4 vs. 48.5 years, *p* = 0.54) and PAA (− 6.7 vs. -7.5, *p* = 0.24) was slightly higher and PAA slightly less in PLWH although this did not reach statistical significance. In multivariate analysis, male sex (adjusted odds ratio = 1.68 [95%CI = 1.03–2.73]), current smoking (2.74 [1.30–5.79]), diabetes mellitus (2.97 [1.48–5.99]), hypertension (1.67 [1.02–2.72]), frailty (3.82 [1.33–10.93]), and higher IL-6 levels (1.09 [1.04–1.15]), but not HIV status and NCI, were independently associated with higher PAA. PhenoAge marker discriminated frailty better than chronological age alone (AUROC: 0.75 [0.66–0.85] vs. 0.65 [0.55–0.77], *p* = 0.04). In the analysis restricted to PLWH, PhenoAge alone predicted frailty better than chronological age alone (AUROC: 0.7412 vs. 0.6499, *P* = 0.09) and VACS index (AUROC: 0.7412 vs. 0.6811, *P* = 0.34) despite not statistically significant.

**Conclusions:**

While PLWH did not appear to have accelerated aging in our cohort, the phenotypic aging marker was significantly associated with systemic inflammation, frailty, and cardiovascular disease risk factors. This simple aging marker could be useful to identify high-risk PLWH within a similar chronological age group.

**Supplementary Information:**

The online version contains supplementary material available at 10.1186/s12877-022-03720-1.

## Introduction

People living with HIV (PLWH) have increased rates of age-related comorbidities compared to the general population [1, 2]. Aging characteristics are heterogeneous among PLWH [3, 4], therefore, the identification of risk factors related to adverse outcomes with aging including cardiovascular diseases, neurocognitive impairment (NCI) and frailty has become increasingly relevant for the older PLWH population.

Recent studies have demonstrated that a novel aging marker “PhenoAge”, which is composed of age, indicators from complete blood count, metabolic markers such as glucose and albumin, markers of organ injury including liver, kidney, and systemic inflammatory marker, can facilitate the identification of at-risk individuals for all-cause mortality, cause-specific mortality, physical functioning, and cognitive performance measures among individuals with similar chronological age [5, 6]. Potential linkages between the PhenoAge acceleration and frailty in the older PLWH population have also been found [7]. Previous studies have demonstrated that Veterans Ageing Cohort Study (VACS) index is also strongly predictive of mortality among PLWH [8, 9]. The difference between PhenoAge and VACS index is that the former was derived from the general population, and it included metabolic and inflammatory markers including glucose, albumin and high sensitivity C-reactive protein (hs-CRP), and aging components such as red cell distribution width and mean cell volume [10], which can better predict age-related comorbidities. However, it is unknown whether PhenoAge can be used as an aging marker for identification of at-risk individuals in the context of HIV.

Previous reports have provided evidence of biological age acceleration in both ART-naïve and treated PLWH compared to HIV-negative people [11–14]. It was also reported that accelerated biological aging was associated with higher risk of HIV-associated neurocognitive disorders (HAND) [15]. However, these studies used epigenetic aging markers derived from DNA methylation (DNAm) techniques to measure the accelerated aging, markers that are not readily available in clinical settings. Despite comorbidities including cardiovascular diseases happening at a younger age among PLWH compared to the general population [16, 17], there is limited availability of useful tools to evaluate such accelerated aging.

In this study, we investigated the phenotypic aging of PLWH and HIV-negative individuals using a simple novel aging biomarker based on routine laboratory measurements in clinical settings. We also evaluated the factors associated with phenotypic age acceleration using an aging cohort study among a group of individuals older than 50 years in Bangkok, Thailand.

## Materials and methods

### Study population

This is a cross-sectional study among PLWH recruited for an aging study cohort at the HIV Netherlands Australia Thailand Research Collaboration (HIV-NAT), Thai Red Cross AIDS Research Centre in Bangkok, Thailand during 2016 and 2018. The characteristics of the aging cohort were described previously [18, 19]. Briefly, people with abnormal fat distribution, such as Cushing syndrome, hypercortisolism and those who were on steroids were excluded. Participants with active opportunistic infections or immunocompromised conditions, such as those receiving chemotherapy were also excluded. PLWH were followed at least every 6 months or annually for HIV care. HIV-negative individuals attending an outpatient clinic at King Chulalongkorn Memorial Hospital were included. HIV-negative controls were matched 1:4 by age (in 5-year interval) and sex with PLWH. Both PLWH and HIV-negative individuals older than 50 years who had been evaluated for the availability of the variables in calculating phenotypic aging marker (defined as below) within +/− 6 months of their cohort enrollment were eligible for this analysis. All methods and study procedures followed relevant regulations and guidelines. All participants provided informed consents and IRB approval was granted for the study.

### Study outcomes and variables

The main study objective was to compare Phenotypic Age (PhenoAge) and Phenotypic age acceleration (PAA) between PLWH and HIV-negative controls. PhenoAge was calculated using chronological age and 9 chemistry biomarkers routinely available at HIV care in low- to middle-income settings, including serum albumin, creatinine, fasting blood glucose, hs-CRP, lymphocyte percentage, mean red cell volume, red cell distribution width, alkaline phosphate, and white cell count [5, 6]. PAA was calculated from the residual resulting from the models when regressing PhenoAge on chronological age, with a negative difference indicating that a participant is phenotypically younger than the chronologic age would suggest.

The Frailty phenotype was assessed using five criteria: unintentional weight loss (≥ 4.5 kg or ≥ 5% of weight loss in the last year or ≥ 2.3 kg or ≥ 2.5% weight loss in last 6 month), low physical activity, self-reported exhaustion (using two questions from the Center for Epidemiologic Studies Depression scale), weak grip strength (two consecutive times on both dominant and non-dominant hands) and slow walking time (on 4-m walk). Using Fried criteria, frailty phenotype was then categorized into robust, pre-frail and frail groups for both PLWH and HIV-negative participants [20]. All the laboratory measurements used to calculate PhenoAge were conducted at the same time of frailty assessment.

The Veterans Aging Cohort Study Index (VACS Index) version 1 was calculated as previously described [9]. Neurocognitive evaluation was completed using the Thai language version of the Montreal Cognitive Assessment (MoCA) which has been validated in HIV infection [21, 22]. NCI was defined as MoCA score ≤ 24. We included socio-demographic variables: educational level, employment status and income level. Other laboratory and clinical data included were body mass index (BMI), comorbidities (diabetes, hypertension, dyslipidemia, cardiovascular diseases); medications including current statin use, oral hypoglycemic or anti-hypertensive agents; serum cholesterol, aspartate aminotransferase (AST) and alanine aminotransferase (ALT), alkaline phosphatase, creatinine, estimated glomerular filtration rate (eGFR) using CKD-EPI [23], serum insulin, hsCRP, interleukin-6 (IL-6) and glucose levels, smoking, alcohol drinking, illicit drug use including methamphetamine and other injecting drugs (evaluated using ASSIST [Alcohol, Smoking and Substance Involvement Screening Test] questionnaire), and hepatitis B and C co-infection status were included. HIV-related variables included in the study were HIV mode of transmission, nadir CD4 and CD8 cell count, current CD4 and CD8 cell count, CD4/CD8 ratio, HIV-1 RNA levels, duration of antiretroviral therapy (ART) and types of ART (non-nucleoside reverse transcriptase inhibitors [NNRTI], protease inhibitors [PI], integrase strand transfer inhibitors [INSTI] and others).

### Statistical analysis

We compared phenotypic age and PAA between PLWH and HIV-negative participants. Continuous variables are presented in mean (±SD, standard deviation) or median (interquartile range [IQR]), and categorical variables as frequencies and percentages. Comparisons between high PAA and low PAA values were made by using Student’s t tests or Wilcoxon rank-sum tests for continuous variables, and a chi-square or Fisher’s exact test for categorical variables. Multivariate logistic regression models were used to investigate the predictors of ‘high PAA’, defined by the value above the median PAA of all participants. Predictors of PhenoAge were investigated using linear regression (log-transformed due to skewed data) and logistic regression for high PhenoAge (defined as higher than the median values) (results presented in Supplementary Tables S2, S3 and S4). The variables in the univariate models with *p*-value < 0.1 were included in the multivariate model.

PhenoAge and PAA were also compared between participants with frailty and those without frailty, stratified by HIV status. Area under observer characteristic curve (AUROC) statistics were used to compare the predictability of frailty using PhenoAge and chronological age and included VACS index in PLWH only model. Since the PhenoAge calculation was originally derived from the general population, a separate analysis was done to compare the ability of PhenoAge and chronological age to predict frailty in PLWH. All analyses were performed using Stata version 16.1 (StataCorp, College Station, Texas 77,845 USA).

## Results

### Phenotypic aging (PhenoAge) and phenotypic age acceleration (PAA)

A total of 435 participants (333 PLWH and 102 HIV-negative controls) were included. 270 (62%) were male and the median age was 55 (IQR, 52–60 years). The overall phenotypic age calculated using the aging marker, PhenoAge was 49.2 years and 48.5 years for PLWH (*P* = 0.54). PhenoAge was moderately correlated with chronological age in both PLWH and HIV-negative participants (Supplementary Fig. S1).

Participants in lower PAA group had similar chronological age as high PAA group (54.8 [IQR, 52.0–58.1] vs. 54.6 [52.1–60.0] *p* = 0.38). Table 1 and table 2 show the differences in characteristics between higher and lower PAA groups. Although statistically not significant, PLWH had slightly greater PAA than HIV-negative participants (− 6.7 vs. -7.5, *p* = 0.24) (Supplementary Table S1).

**Table 1:** Characteristics of all participants by low and high phenotypic age acceleration (PAA)

Total ***N*** = 435	Low PAA (***N*** = 218)	High PAA (***N*** = 217)	***P***-value
**Age**	54.8 (52.0–58.1)	54.6 (52.1–60.0)	0.42
**Sex**			< 0.001
Male	114 (52.29)	156 (71.89)	
Female	104 (47.71)	61 (28.11)	
**Body mass index** (BMI), kg/m^2^	23.2 (20.8–25.4)	23.7 (21.4–26.2)	0.11
**Waist circumference**, cm	83.5 (77–90)	86 (80–92)	0.004
**HIV-positive**	160 (73.39)	173 (79.72)	0.119
**Education**			0.71
No Education	3 (1.38)	1 (0.46)	
Primary/Secondary school	91 (41.74)	85 (39.17)	
High School / Vocational	59 (27.06)	65 (29.95)	
Higher than bachelor’s degree	65 (29.82)	66 (30.41)	
**Employment**			0.30
Unemployed	38 (17.43)	30 (13.82)	
Employed	180 (82.57)	187 (86.18)	
**Income group (THB, monthly)**			0.17
No income	23 (10.55)	16 (7.37)	
< 10,000	66 (30.28)	51 (23.5)	
10,000-19,999	58 (26.61)	72 (33.18)	
> =20,000	71 (32.57)	78 (35.94)	
**Low physical activity**			0.030
No	147 (73.87)	127 (63.82)	
Yes	52 (26.13)	72 (36.18)	
**Smoking**			< 0.001
Never	159 (72.94)	122 (56.22)	
Ex-smoker	43 (19.72)	55 (25.35)	
Current	16 (7.34)	40 (18.43)	
**Alcohol drinking**			0.001
Never	183 (83.94)	154 (70.97)	
Ex-drinker	15 (6.88)	38 (17.51)	
Current	20 (9.17)	25 (11.52)	
**Illicit drug use**			
Ever use	4/210 (1.9)	2/210 (0.95)	0.685
Current use	2/210 (0.95)	1/210 (0.48)	1.00
**HBV infection**	22 (10.09)	26 (11.98)	0.53
**HCV infection**	17 (7.8)	16 (7.37)	0.87
**Diabetes mellitus**	16 (7.34)	55 (25.35)	< 0.001
**Hypertension**	63 (28.9)	104 (47.93)	< 0.001
**Cardiovascular diseases (CVD)**	6 (2.75)	12 (5.53)	0.16
**Current statin use**	55 (25.23)	73 (33.64)	0.054
**Total cholesterol**, mg/dL	209.5 (186–243)	207 (180–240)	0.30
**HDL-cholesterol**, mg/dL	49 (41–60)	46 (39–54)	0.003
**LDL-cholesterol**, mg/dL	128.2 (104–155)	124(101–148)	0.17
**Triglycerides**, mg/dL	140 (95–189)	153 (106–224)	0.017
**Creatinine**, mg/dL	0.82 (0.72–0.95)	0.91 (0.8–1.09)	< 0.001
**eGFR (CKD-EPI)**, mL/min/1.73m^2^	93.95 (83.93–99.21)	85.31 (71.28–97.02)	< 0.001
**ALT**, IU/mL	26 (20–35)	29 (21–41)	0.037
**AST**, IU/mL	25 (20–30)	25 (20–32)	0.46
**ALP**, IU/mL	3.4 (3.1–3.7)	3.2 (2.9–3.7)	0.003
**APRI**, IU/mL	0.24 (0.19–0.32)	0.24 (0.18–0.33)	0.88
**FIB-4 score**	1.03 (0.85–1.3)	0.98 (0.79–1.35)	0.47
**Albumin**	43 (41–46)	42 (38–45)	< 0.001
**Insulin**, IU/mL	5.7 (3.9–8.5)	7.2 (4.9–11.2)	0.0010
**hs-CRP**, mg/dL	0.09 (0.05–0.19)	0.17 (0.08–0.34)	< 0.001
**IL-6**, pg/mL	5.27 (3.57–6.83)	6.92 (5–11.42)	< 0.001
**Montreal Cognitive Assessment (MOCA)**	24 (21–26), n = 218	23 (21–26), *n* = 216	0.29
Abnormal MOCA (≤24)	131 (60.09)	134 (62.04)	0.68
**Frailty status**			0.001
Robust	86 (43.22)	72 (36.18)	
Pre-frail	107 (53.77)	102 (51.26)	
Frail	6 (3.02)	25 (12.56)	

**Abbreviations**: *PAA* phenotypic age acceleration, *CKD-EPI* Chronic Kidney Disease-Epidemiology Collaboration, *AST* aspartate transaminase, *ALT* alanine aminotransferase, *ALP* alkaline phosphatase, *hs-CRP* high-sensitive C-reactive protein

**Table 2:** Characteristics of PLWH participants by low and high phenotypic age acceleration (PAA)

HIV-related parameters (n = 333)	Low PAA (***N*** = 160)	High PAA (***N*** = 173)	P-value
**Mode of HIV acquisition**			0.92
Heterosexual	119 (74%)	126 (73%)	
Homosexual	18 (11%)	24 (14%)	
Injecting drug use	1 (1%)	1 (1%)	
Other	3 (2%)	2 (1%)	
Unknown	19 (12%)	20 (11%)	
**Nadir CD4 count** (cells/mm^3^)	178 (88–257)	175 (92–242)	0.64
**Current CD4 count** (cells/mm^3^)	664 (511–819)	596 (441–769)	0.024
**Current CD8 count** (cells/mm^3^)	656 (516–846)	726 (517–950)	0.18
**Nadir CD8 count** (cells/mm^3^)	428 (301–554)	435 (298–646)	0.37
**Current CD4:CD8 ratio**	1.00 (0.74–1.33)	0.88 (0.63–1.22)	0.028
**Baseline HIV-1 RNA** (log_10_ copies/mL)	*n* = 114	*n* = 103	0.18
	4.74 (4.33–5.24) *n* = 114	4.69(4.13–5.11) *n* = 103	
**Current undetectable HIV-1 RNA** (< 50 copies/mL)	157 (98%)	168 (97%)	0.73
**CDC staging at baseline**			0.75
A	74 (46%)	73 (42%)	
B	62 (38%)	73 (42%)	
C	24 (15%)	27 (16%)	
**CDC staging at current**			0.83
A	63 (40%)	63 (37%)	
B	70 (44%)	81 (47%)	
C	25 (16%)	28 (16%)	
**Duration of ART** (years)	16 (12–19)	16 (14–19)	0.35
**Duration of HIV** (years)	18 (15–21)	19 (16–21)	0.23
**Current ART regimen**			0.048
NNRTI	102 (64%)	83 (48%)	
PI	38 (24%)	63 (36%)	
NNRTI+PI	10 (6%)	11 (6%)	
INSTI	2 (1%)	5 (3%)	
Other	8 (5%)	11 (6%)	
**VACS index score**	22 (12–28)	22 (18–34)	0.012

Socio-economic characteristics such as education level, employment status, and monthly income levels were not significantly different between the groups. Levels of neurocognitive impairment assessed by MOCA was similar (*p* = 0.29), however, the burden of comorbidities such as hypertension, diabetes mellitus and CVD history were more prevalent in high PAA group. Individuals in high PAA group also had higher proportion of low physical activity (36.2% vs. 26.1%, *p* = 0.03). In addition, systemic inflammatory marker, IL-6, was also higher in high PAA group (*p* < 0.001). Additionally, 13% of high PAA group were frail compared to 3% of low PAA group (p < 0.001).

Proportions of PLWH between the two PAA groups were not statistically different (73% vs. 80%, *p* = 0.12). Among PLWH, high PAA group had lower current CD4 cell count (596 [IQR, 441–769] vs. 664 [511–819] cells/mm^3^, *p* = 0.02) and lower CD4/CD8 ratio (0.88 [0.63–1.22] vs. 1.00 [0.74–1.33], *p* = 0.03) than low PAA group. Although the two groups had similar medians for the VACS index score, the rank was significantly different and higher for high PAA group compared to low PAA group using Wilcoxon test (*p* = 0.01) (Supplementary Fig. S2).

**Fig. 1:**
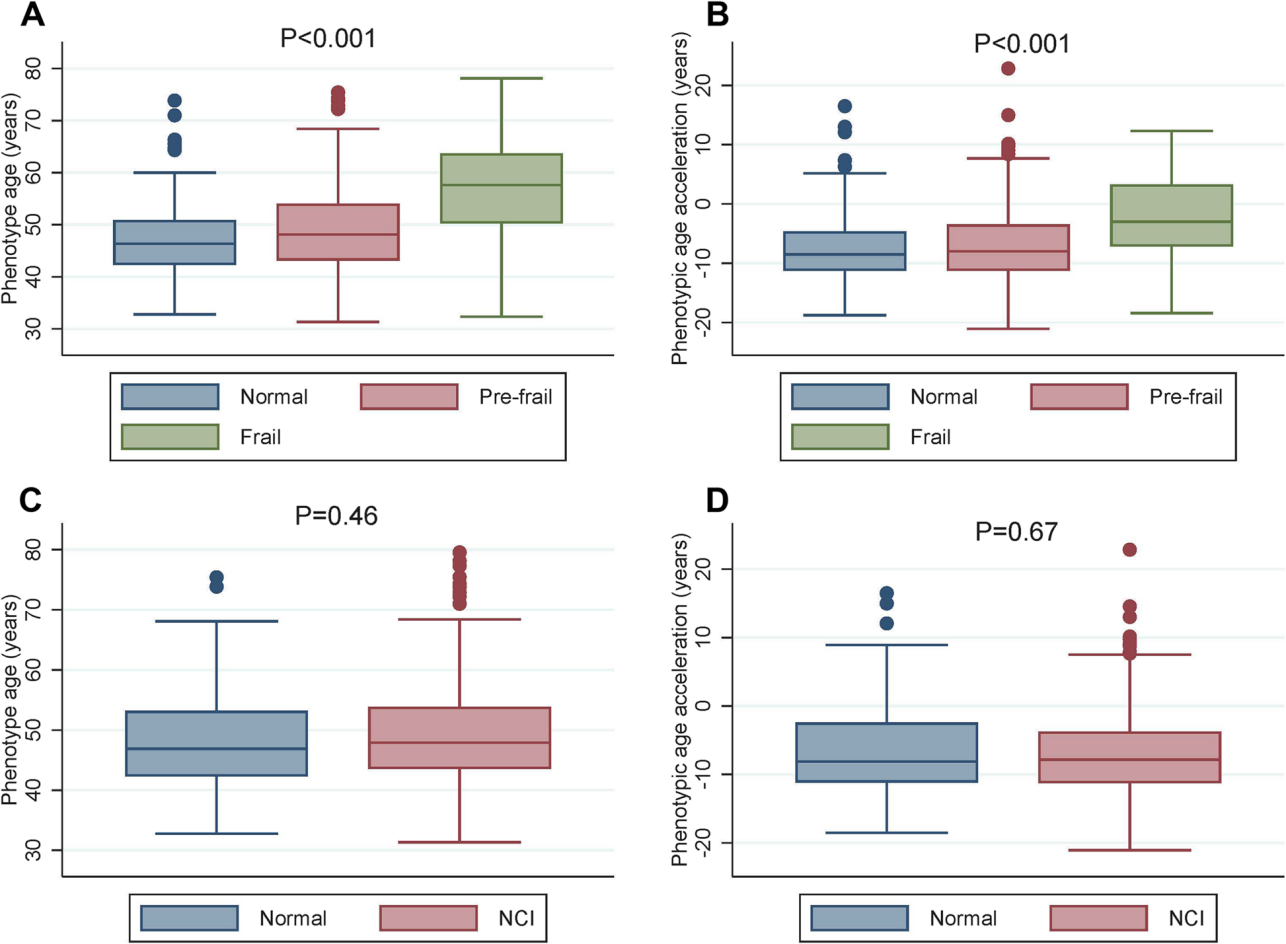
Comparison of PhenoAge and PAA between frailty and NCI status in all population. **A** and **B** show the differences in PhenoAge and PAA with frailty status, respectively (*P* values < 0.001). PhenoAge and PAA were not statistically different between individuals with and without NCI in all population

**Fig. 2:**
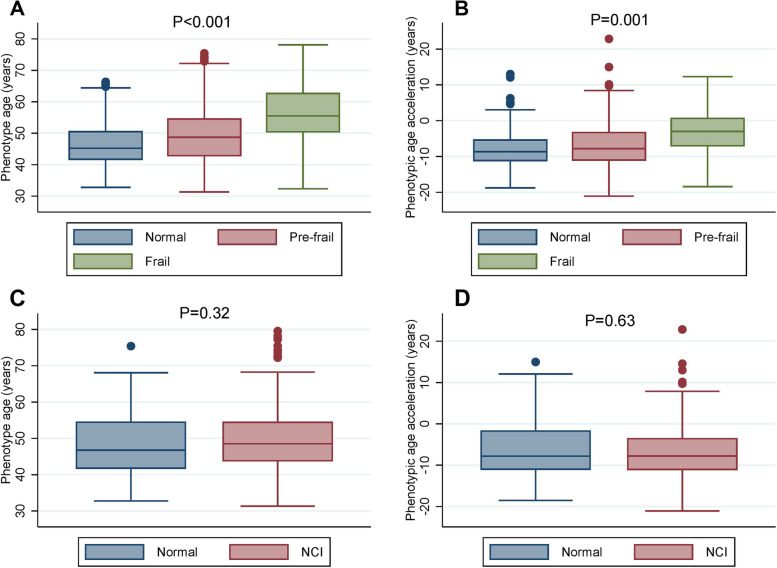
Comparison of PhenoAge and PAA between frailty and NCI status in PLWH only. **A** and **B** show the differences in PhenoAge and PAA with frailty status in PLWH, respectively (*P* < 0.001 for PhenoAge and *P* = 0.001 for PAA). PhenoAge and PAA were not statistically different between individuals with and without NCI in PLWH

Figures 1 and 2 show that frailty group has higher PhenoAge (*p* < 0.001) and PAA (p < 0.001) than normal and pre-frail groups overall and among PLWH. However, PhenoAge and PAA were not statistically different between individuals with or without NCI (*p* > 0.2). Individuals with higher disease counts had significantly higher PhenoAge and PAA among both HIV-negative and HIV-positive groups (Supplementary Fig. S3).

### Factors associated with high PAA in all participants (*N* = 435)

In a multivariate logistic regression model among all participants, male sex (adjusted odds ratio [aOR] = 1.68, 95% CI 1.03, 2.73, *p* = 0.039), current smoking (aOR = 2.74, 95% CI 1.30, 5.79, *p* = 0.008; compared to never or ex-smoker), diabetes mellitus (aOR = 2.97, 95% CI 1.48, 5.99, *p* = 0.002), hypertension (aOR = 1.67, 95% CI 1.02, 2.72, *p* = 0.04), higher IL-6 level (aOR = 1.09, 95% CI 1.04, 1.15, *p* = 0.001), and frailty (aOR = 3.82, 95% CI 1.33, 10.93, *p* = 0.012) were significantly associated with high PAA (Table 3). We did not find a significant association of HIV status with high PAA in the multivariable model (aOR = 1.18, 95% CI 0.68, 2.04, *p* = 0.56).

**Table 3:** Univariate and multivariate logistic regression for high PAA in all participants

All participants	Univariate			Multivariate		
	OR	95% CI	*p*-value	aOR	95% CI	*p*-value
**Male** (vs. female)	2.33	1.57, 3.47	< 0.001	**1.68**	**1.03, 2.73**	**0.039**
**BMI** ≥ 25 kg/m^2^ (vs. < 25 kg/m^2^)	1.35	0.90, 2.02	0.143			
**HIV status**	1.43	0.91, 2.23	0.12	1.18	0.68, 2.04	0.56
**Waist circumference,** cm (per unit increase)	1.03	1.01, 1.05	0.006	1.00	0.97, 1.02	0.70
**Smoking**						
Never/ ex-smoker	Ref					
current	2.85	1.54, 5.27	0.001	**2.74**	**1.30, 5.79**	**0.008**
**Alcohol drinking**						
Never/ ex-smoker	Ref					
current	1.29	0.69, 2.40	0.423			
**Diabetes mellitus**	4.29	2.37, 7.76	< 0.001	**2.97**	**1.48, 5.99**	**0.002**
**Hypertension**	2.26	1.52, 3.36	< 0.001	**1.67**	**1.02, 2.72**	**0.040**
**Statin use**	1.50	0.99, 2.28	0.055	1.04	0.62, 1.73	0.88
**HDL-cholesterol** ≤40 mg/dL (vs. > 40 mg/dL)	1.49	0.97, 2.27	0.065			
**LDL-cholesterol** ≥130 mg/dL (vs. < 130 mg/dL)	0.86	0.58, 1.25	0.423			
**Triglycerides** ≥150 mg/dL (vs. < 150 mg/dL)	1.31	0.90, 1.90	0.17			
**ALT**, IU/mL (per unit increase)	1.01	1.00, 1.02	0.035	1.01	1.00, 1.02	0.10
**Insulin**, IU/mL (per unit increase)	1.05	1.02, 1.08	0.001			
**IL-6**, pg/ml (per unit increase)	1.10	1.05, 1.15	< 0.001	**1.09**	**1.04, 1.15**	**0.001**
**Frailty status**						
Normal	Ref			Ref		
Pre-frailty	1.14	0.75, 1.72	0.539	1.05	0.66, 1.67	0.83
Frailty	4.98	1.94, 12.8	0.001	**3.82**	**1.33, 10.93**	**0.012**

* Diabetes mellitus was defined as fasting blood glucose ≥126 mg/dL for two consecutive visits of 6 months interval or a physician diagnosis or taking anti-diabetes medications. Hypertension was defined as SBP ≥140 mmHg or DBP ≥90 mmHg for two consecutive visits of 6 months interval or taking anti-hypertensive medications

Abbreviations: *aOR* adjusted odds ratio, *BMI* body mass index, *LDL* low-density lipoprotein, *HDL* high-density lipoprotein, *ALT* alanine aminotransferase, *IL-6* interleukin-6

### Factors associated with high PAA in PLWH participants (*N* = 333)

In models restricted to PLWH and adjusted for either current CD4 count (model 1) or current CD4/CD8 ratio (model 2), diabetes mellitus, current smoking, higher IL-6 level, and frailty were associated with high PAA (Table 4) after adjusting for types of antiretrovirals (NNRTI, PI and others) and duration of ART. We also did not find the specific antiretrovirals such as TDF or protease inhibitors associated with high PAA.

**Table 4:** Univariate and Multivariate logistic regression for high PAA among PLWH participants

PLWH participants	Univariate			Multivariate 1			Multivariate 2		
	OR	95% CI	*p*-value	aOR	95% CI	*p*-value	aOR	95% CI	*p*-value
**Male**	2.00	1.28, 3.14	0.002	1.26	0.72, 2.22	0.42	1.36	0.78, 2.37	0.28
**BMI** ≥ 25 kg/m^2^ (vs. < 25 kg/m^2^)	1.48	0.90, 2.42	0.119	1.07	0.58, 1.98	0.82	1.07	0.58, 1.97	0.84
**Abnormal waist circumference**	1.17	0.76, 1.81	0.48						
**Smoking**									
Never/ ex-smoker	Ref								
current	2.57	1.29, 5.09	0.007	**3.04**	**1.18, 7.81**	**0.021**	**2.70**	**1.09, 6.65**	**0.031**
**Alcohol drinking**									
Never/ ex-smoker	Ref								
current	1.96	0.89, 4.33	0.096	1.61	0.54, 4.78	0.39	1.70	0.58, 4.94	0.33
**Diabetes mellitus**	4.08	2.06, 8.07	< 0.001	**2.88**	**1.28, 6.51**	**0.011**	**2.84**	**1.26, 6.41**	**0.012**
**Hypertension**	1.89	1.21, 2.94	0.005	1.36	0.79, 2.35	0.27	1.43	0.83, 2.47	0.20
**Statin use**	1.50	0.94, 2.38	0.086	1.24	0.7, 2.17	0.46	0.48	0.70, 2.15	0.48
**HDL-cholesterol** ≤40 mg/dL (vs. > 40 mg/dL)	1.24	0.78, 1.98	0.355						
**LDL-cholesterol** ≥130 mg/dL (vs. < 130 mg/dL)	0.90	0.58, 1.41	0.656						
**Triglycerides** ≥150 mg/dL (vs. < 150 mg/dL)	1.30	0.85, 2.01	0.228						
**ALT** > 50	1.09	0.61, 1.95	0.778						
**Insulin**, IU/mL (per unit increase)	1.05	1.02, 1.08	0.004						
**IL-6**, pg/ml (per unit increase)	1.12	1.06, 1.18	< 0.001	**1.11**	**1.04, 1.18**	**0.001**	**1.11**	**1.04, 1.18**	**0.002**
**Current CD4** ≥ 500 cells/mm^3^ (vs. < 500 cells/mm^3^)	0.60	0.37, 0.97	0.039				0.68	0.38, 1.20	0.18
**Current CD4/CD8 ratio** ≥ 1 (vs. < 1)	0.60	0.38, 0.95	0.029	0.68	0.39, 1.19	0.18			
**Baseline HIV-1 RNA** > 5 log_10_ copies/mL (vs. ≤5 log_10_ copies/mL)	0.77	0.44, 1.36	0.371						
**ART class**									
NNRTI	Ref			Ref					
PI	2.04	1.241, 3.35	0.005	1.54	0.85,2.79	0.15	1.61	0.89, 2.90	0.11
Other	1.66	0.87, 3.17	0.125	1.07	0.48,2.39	0.86	1.12	0.51, 2.47	0.78
**Duration of ART** ≥ 10 years (vs. < 10 years)	0.75	0.37, 1.51	0.42						
**Frailty status**									
Normal	Ref								
Pre-frailty	1.45	0.89, 2.37	0.139	1.27	0.72,2.21	0.41	1.33	0.76, 2.32	0.32
Frailty	5.11	1.92, 13.59	0.001	**3.43**	**1.11,10.63**	**0.032**	**3.58**	**1.17, 10.92**	**0.025**

*Abnormal waist circumference was defined as waist circumference > 80 cm in females and > 90 cm in males. Diabetes mellitus was defined as fasting blood glucose ≥126 mg/dL for two consecutive visits of 6 months interval or a physician diagnosis or taking anti-diabetes medications. Hypertension was defined as SBP ≥140 mmHg or DBP ≥90 mmHg for two consecutive visits of 6 months interval or taking anti-hypertensive medications

Abbreviations: *aOR* adjusted odds ratio, *BMI* body mass index, *LDL* low-density lipoprotein, *HDL* high-density lipoprotein, *ALT* alanine aminotransferase, *IL-6* interleukin-6, *ART* antiretroviral therapy, *NNRTI* non-nucleotide reverse transcriptase inhibitor, *PI* protease inhibitor

Model 1 included current CD4 count and model 2 included current CD4/CD8 ratio in the multivariate analyses

In separate analyses using multivariate linear regression, similar predictors were found for PhenoAge as high PAA (Supplementary Tables S2, S3 and S4).

### Discriminative ability of PhenoAge, chronological age and VACS index in identifying frailty and NCI

**Fig. 3:**
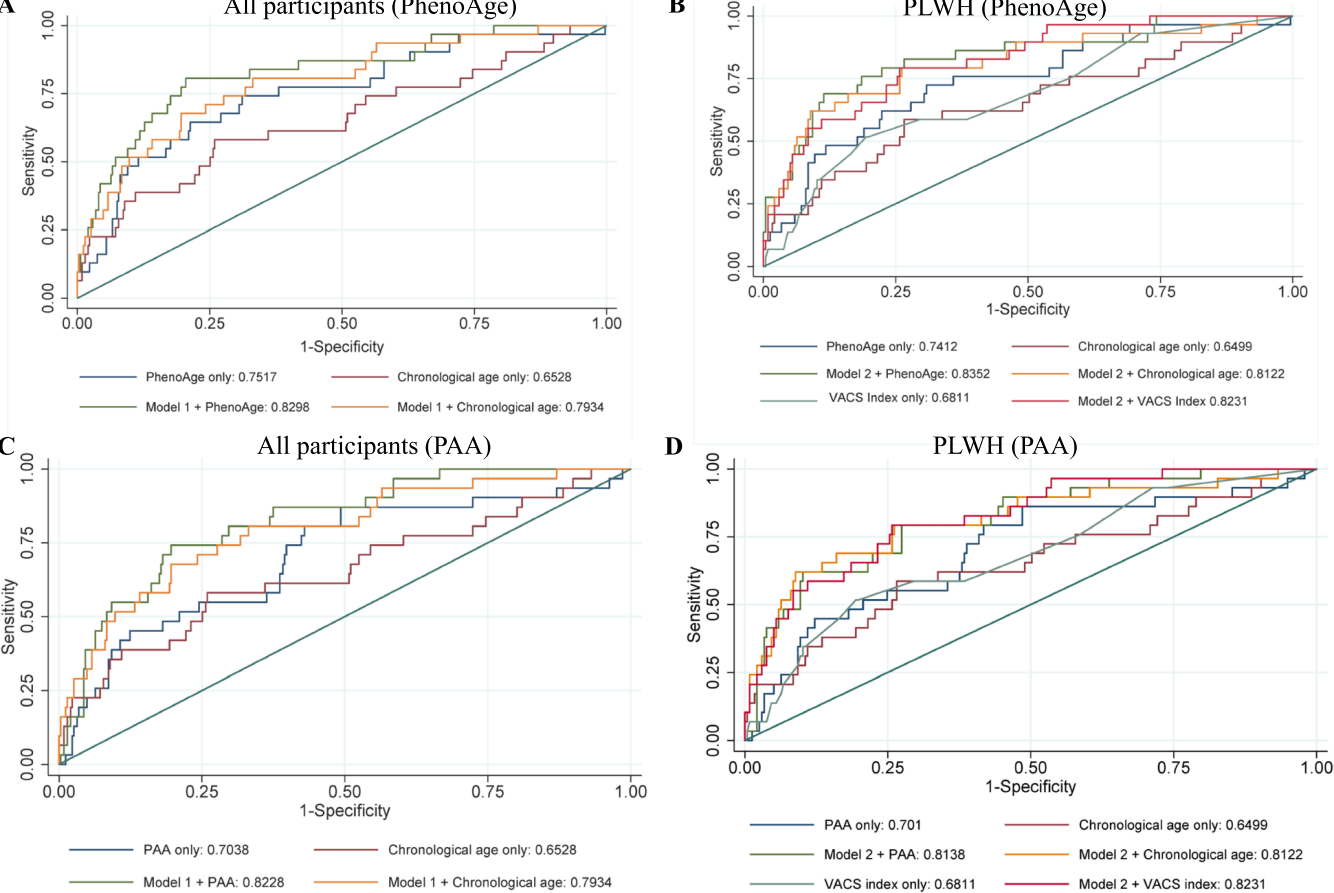
Receiver-operating characteristic curves for frailty in overall (3A) and among PLWH (3B). AUROC curves in **A** shows PhenoAge alone predicted frailty risk better than chronological age alone (AUROC: 0.7517 vs. 0.6528, *P* = 0.040) (**A**). It was also higher in the adjusted analysis (model 1) (AUROC: 0.8298 vs. 0.7934, *P* = 0.104) although statistically not significant. In the analysis restricting to PLWH, AUROC curves in **B** shows PhenoAge alone predicted frailty better than chronological age alone (AUROC: 0.7412 vs. 0.6499, *P* = 0.09) and VACS index (AUROC: 0.7412 vs. 0.6811, *P* = 0.34) although it was statistically significant. Model 2 + PhenoAge has significantly better discriminative ability for frailty compared with VACS index alone (AUROC: 0.835 vs. 0.6811, *P* = 0.02), but not with model 2 + VACS index (AUROC: 0.835 vs. 0.8231, *P* = 0.73). **C** and **D** show PAA has lower AUROC value compared to PhenoAge in predicting frailty risk among all participants and PLWH. In **D**, PAA predicted similarly as chronological age and VACS index in the adjusted models (i.e., *P* > 0.1 for AUROC of model 2 + chronological age or model 2 + VACS index compared to model 2 + PAA)/ Model 1 was adjusted for sex, BMI, smoking, alcohol drinking, education level, income, diabetes mellitus, hypertension, and statin use. Model 2 was adjusted for the variables in model 1 + current CD4 count and duration of ART

Figure. 3A shows that PhenoAge predicted frailty better than chronological age (AUROC: 0.76 [95%CI: 0.66–0.85] vs. 0.66 [95%CI: 0.55–0.77], *p* = 0.04). In models restricted to PLWH, we found similar discriminative abilities though no longer reached statistical significance in the frailty model (Fig. 3B) Similar findings were observed for PAA in predicting frailty risk although AUROC values were slightly lower than PhenoAge (AUROC: 0.70 for PAA vs. 0.65 for chronological age, *p* = 0.04) (Fig. 3C). However, in the analysis restricted to PLWH, PAA predicted frailty similarly as the VACS Index and chronological age (*P* > 0.1) (Fig. 3D).

The discriminative ability to detect NCI did not differ by PhenoAge and chronological age in combined models of PLWH and HIV-negative participants (*p* > 0.1), or models restricted to PLWH. AUROC for NCI did not differ between PhenoAge, VACS index and chronological age among PLWH (*p* > 0.2).

## Discussion

In this cross-sectional study, we compared PhenoAge and PAA among PLWH and HIV-uninfected controls. Although our study failed to find significant differences by HIV serostatus, we did find that greater PAA was associated with male sex, smoking, systemic inflammation, frailty, and comorbidities, including diabetes and hypertension. Among PLWH, current CD4 cell count and CD4/CD8 ratio were higher in high PAA group compared to low PAA group. We also observed that PhenoAge better discriminates frailty than chronological age or the VACS Index although the difference was slightly reduced in the analysis restricted to PLWH only and this was not statistically different for PAA compared to chronological age or the VACS Index. We did not find the pronounced effect of types of antiretrovirals, duration of ART and other HIV-related characteristics on PAA of older PLWH with long-term suppression. However, our study results highlighted the point that PLWH are aging heterogeneously and PhenoAge could be used as a clinical tool to identify high-risk individuals.

To our knowledge, this is the first study to evaluate, among aging population living with HIV, the novel blood chemistry-based aging marker (PhenoAge) which has been shown to be able to identify the high-risk individuals for all-cause mortality and comorbidities in the general population. Previous studies have shown that epigenetic age acceleration was greater in untreated ART-naïve adults with HIV than HIV-negative controls [14], and still elevated after 2-years of ART initiation [14]. However, in our study population of long-term ART-experienced older PLWH with viral suppression, HIV infection or duration of HIV was not associated with significant epigenetic age acceleration. This could be explained by the possibility that PAA was driven more pronouncedly by chronic inflammation, comorbidities, and other immunological characteristics. It is also possible that PhenoAge of PLWH have improved over the years of ART. However, our study could not identify such longitudinal recovery of epigenetic aging.

A previous study with small sample size (*N* = 10) has shown that epigenetic aging, evaluated using DNAm methods, was accelerated in PLWH with chronic infection who were frail compared to those who were not [7]. Frailty represents an increased physiologic vulnerability with aging, often associated with increased comorbid burden, higher inflammation, markers of immune activation and immune dysfunction in PLWH. Thus, the association between frailty and PAA found in our study is novel, but not surprising. Whether higher PAA has add-on effects on frailty in predicting the worse prognosis among PLWH population, or whether PAA might act as a brief tool to identify frail patients for further clinical evaluation are important unanswered questions.

In a large study evaluating a different aging phenotypic marker, epigenetic aging was associated with smoking and immunosenescence, including negative correlation with naïve CD8+ T cell, naïve CD4+ T cell and leukocyte telomere length, and positive correlation with exhausted CD8+ T cells [5, 24]. The association of CD4/CD8 ratio, which is used as surrogate marker for immunosenescence and immune dysfunction [25, 26], with high PAA is also observed in our study. In addition to smoking status, PhenoAge was associated with physical functioning status among both smokers and non-smokers in the general population [5]. Our study results among PLWH are consistent with those findings: higher proportion of individuals with low physical activity was found in high PAA group in this study. Moreover, higher VACS index, plasma IL-6 level and low CD4/CD8 ratio were the high PAA group in our study. This indicates that individuals with high PAA could also be at higher risks for activated inflammation and hence comorbidities and adverse clinical outcomes [27]. A recent paper has also demonstrated that DNAm-based age acceleration markers were associated with increased VACS index and mortality [28].

A previous study showed that HIV infection increases the epigenetic aging in brain tissue [29]. Another study also demonstrated that HAND in a group of PLWH participants was associated with accelerated aging measured through epigenetics, with average acceleration of 3.5 years, compared to neurocognitively healthy people [15]. A recent study using different DNAm-based biomarkers also demonstrated that DNAm-based PhenoAge was associated with executive function and attention [30]. In contrast to our study with blood chemistry-based PhenoAge, we did not find the association of accelerated aging and NCI assessed using MoCA. The lack of significant association of NCI with high PAA could be explained by the screening tool used in our study. Although MoCA has good sensitivity and specificity for initial screening of mild to moderate cognitive impairment among PLWH, its performance among older PLWH is moderate or limited [18, 31]. However, the association of increased PAA with frailty and multiple age-related comorbidities suggests that this epigenetic aging biomarker which can be easily calculated from clinically available laboratory parameters and relatively cheaper compared to DNAm methods, could be a useful tool to screen for high-risk individuals, and those at increased risks might benefit from potential targeted interventions.

Our study has several limitations to be acknowledged. Firstly, due to its cross-sectional observational study design, we were not able to evaluate the changes of PhenoAge or PAA over time. Although we have tried to include all available socio-demographic variables, residual confounding effects from other lifestyle factors such as diet and exercise and co-infection such cytomegalovirus infection cannot be ruled out. Secondly, majority of our PLWH participants were virally suppressed, and therefore, we were not able to evaluate the effect of HIV viremia on PAA. Finally, our older PLWH were on long-term ART and the impacts of ART on PhenoAge marker were not able to be assessed. Nonetheless, this novel aging measure which include multiple aspects of laboratory measurements could provide an important tool for evaluating efficacy from interventional measures in studies of aging-related comorbidities.

## Conclusions

Our study shows that the novel aging measure, calculated from routinely available laboratory measurements from HIV clinics, was associated with frailty, systemic inflammation, and multiple age-related comorbidities among older PLWH. It could serve as a useful tool to evaluate the heterogeneity of aging and to identify the at-risk population. Further studies are needed to confirm its usefulness in more diverse population in other cohorts and its longitudinal relationship with other comorbidities such as malignancies and mortality in the context of HIV infection.

## Supplementary Information

**Additional file 1.** Supplementary data.

## Data Availability

The datasets used and/or analyzed during the current study are available from the corresponding author on reasonable request.

## Abbreviations

AUROC: Area under receiver operating characteristic
BMI: Body mass index
VACS: Veterans Aging Cohort Study
PAA: Phenotypic age acceleration
