# Supplementary material for: Association of Phenotypic Aging Marker with comorbidities, frailty and inflammatory markers in people living with HIV
Source: BMC Geriatr. 2022 Dec 31;22:1010. doi: 10.1186/s12877-022-03720-1 (PMC9805022; doi:10.1186/s12877-022-03720-1)
Supplement: Supplementary file 1 — Additional file 1. Supplementary data. [file 12877_2022_3720_MOESM1_ESM.docx]

**Supplementary data**

**Table S1**. Phenotypic age by HIV-status

| **Phenotypic age** | Mean | SD | p50 | p25 | p75 | Min | Max | N |
| --- | --- | --- | --- | --- | --- | --- | --- | --- |
| HIV-Negative | 48.51 | 7.39 | 47.52 | 43.63 | 52.06 | 35.6 | 73.84 | 102 |
| HIV-Positive | 49.39 | 8.93 | 47.83 | 43.03 | 54.63 | 31.34 | 79.55 | 333 |
| Total | 49.18 | 8.6 | 47.82 | 43.17 | 53.52 | 31.34 | 79.55 | 435 |
| p-value= 0.54* |  |  |  |  |  |  |  |  |
| **PAA** |  |  |  |  |  |  |  |  |
| HIV-Negative | -7.48 | 5.84 | -8.47 | -11.42 | -4.77 | -16.34 | 16.49 | 102 |
| HIV-Positive | -6.68 | 6.67 | -7.77 | -11.13 | -3.00 | -21.08 | 22.85 | 333 |
| Total | -6.86 | 6.49 | -7.93 | -11.28 | -3.28 | -21.08 | 22.85 | 435 |
| p-value= 0.24* |  |  |  |  |  |  |  |  |

*p-value by Wilcoxon rank-sum

**Table S2**. Linear regression for “log-transformed” PhenoAge in all participants

| **All participants** | **Univariate** | | | | **Multivariate** | | |
| --- | --- | --- | --- | --- | --- | --- | --- |
|  | **coefficients** | **95% CI** | **p-value** | **coefficients** | | **95% CI** | **p-value** |
| Female | -0.03 | -0.04, -0.01 | <0.001 | -0.02 | | -0.03, -0.01 | **0.004** |
| BMI≥25 | 0.02 | 0.001, 0.03 | 0.034 |  | |  |  |
| HIV status | 0.01 | -0.01, 0.02 | 0.487 | -0.003 | | -0.02,0.01 | 0.69 |
| waist circumference | 0.001 | 0.001, 0.002 | 0.001 | 0.00 | | -0.001,0.001 | 0.81 |
| Smoking |  |  |  |  | |  |  |
| Never/ ex-smoker | Ref |  |  |  | |  |  |
| current | 0.02 | -0.002, 0.04 | 0.07 | 0.01 | | -0.01,0.03 | 0.57 |
| Alcohol drinking |  |  |  |  | |  |  |
| Never/ ex-smoker | Ref |  |  |  | |  |  |
| current | 0.005 | -0.02, 0.03 | 0.685 |  | |  |  |
| Diabetes mellitus | 0.07 | 0.05, 0.09 | <0.001 | 0.05 | | 0.04,0.07 | **<0.001** |
| Hypertension | 0.05 | 0.03, 0.06 | <0.001 | 0.03 | | 0.02, 0.05 | **<0.001** |
| Statin use | 0.02 | 0.004, 0.03 | 0.015 | 0.003 | | -0.01,0.02 | 0.72 |
| HDL-cholesterol<40 | 0.01 | -0.002, 0.03 | 0.09 |  | |  |  |
| LDL-cholesterol≥130 | -0.004 | -0.02, 0.01 | 0.575 |  | |  |  |
| Triglycerides≥150 | 0.01 | -0.00, 0.03 | 0.051 |  | |  |  |
| ALT>50 | 0.01 | -0.01, 0.03 | 0.274 |  | |  |  |
| Insulin | 0.001 | 0.001, 0.002 | 0.001 |  | |  |  |
| IL-6 (log_10_) | 0.06 | 0.04, 0.07 | <0.001 | 0.04 | | 0.02,0.06 | **<0.001** |
| Fragility status |  |  |  |  | |  |  |
| Normal | Ref |  |  |  | |  |  |
| Pre-frailty | 0.02 | 0, 0.03 | 0.037 | 0.01 | | 0.0001,0.03 | **0.048** |
| Frailty | 0.08 | 0.05, 0.11 | <0.001 | 0.06 | | 0.04,0.09 | **<0.001** |
| Diabetes mellitus was defined as fasting blood glucose ≥126 mg/dL for two consecutive visits of 6 months interval or a physician diagnosis or taking anti-diabetes medications. Hypertension was defined as SBP ≥140 mmHg or DBP ≥90 mmHg for two consecutive visits of 6 months interval or taking anti-hypertensive medications.  Abbreviations: aOR, adjusted odds ratio; BMI, body mass index; LDL, low-density lipoprotein; HDL, high-density lipoprotein; ALT, alanine aminotransferase; IL-6, interleukin-6. | | | | | | | |

**Table S3**. Linear regression for “log-transformed” PhenoAge in PLWH participants

| **Only HIV** | **Univariate** | | | | **Multivariate** | | | |
| --- | --- | --- | --- | --- | --- | --- | --- | --- |
|  | **coefficients** | **95% CI** | **p-value** | **coefficients** | | **95% CI** | **p-value** |  |
| Female | -0.03 | -0.05, -0.01 | 0.001 | -0.02 | | -0.04, 0.002 | 0.07 |  |
| BMI≥25 | 0.02 | 0, 0.04 | 0.043 |  | |  |  |  |
| waist circumference | 0.001 | 0.001, 0.002 | 0.001 | 0.00 | | -0.001, 0.001 | 0.83 |  |
| Smoking |  |  |  |  | |  |  |  |
| Never/ ex-smoker | Ref |  |  |  | |  |  |  |
| current | 0.01 | -0.01, 0.04 | 0.283 |  | |  |  |  |
| Alcohol drinking |  |  |  |  | |  |  |  |
| Never/ ex-smoker | Ref |  |  |  | |  |  |  |
| Current | 0.01 | -0.02, 0.04 | 0.552 |  | |  |  |  |
| Diabetes mellitus | 0.07 | 0.05, 0.09 | <0.001 | 0.05 | | 0.02, 0.08 | **0.001** |  |
| Hypertension | 0.05 | 0.04, 0.07 | <0.001 | 0.03 | | 0.02, 0.06 | **0.001** |  |
| Statin use | 0.02 | 0.002, 0.04 | 0.032 | -0.001 | | -0.02, 0.02 | 0.92 |  |
| HDL-cholesterol<40 | 0.01 | -0.01, 0.03 | 0.316 |  | |  |  |  |
| LDL-cholesterol≥130 | -0.002 | -0.02, 0.01 | 0.812 |  | |  |  |  |
| Triglycerides≥150 | 0.01 | -0.002, 0.03 | 0.085 |  | |  |  |  |
| ALT>50 | 0.01 | -0.02, 0.03 | 0.619 |  | |  |  |  |
| Insulin | 0.001 | 0.00, 0.002 | 0.006 |  | |  |  |  |
| IL-6 (log_10_) | 0.06 | 0.04, 0.08 | <0.001 | 0.05 | | 0.02, 0.07 | **<0.001** |  |
| Current CD4 >500 | -0.02 | -0.04, -0.001 | 0.036 | -0.01 | | -0.03,0 .01 | 0.22 |  |
| CD4/CD8 ratio >1 | -0.01 | -0.03, 0.002 | 0.086 |  | |  |  |  |
| Baseline HIV-1 RNA >5 log_10_ copies/mL | -0.02 | -0.04, 0.005 | 0.13 | -0.02 | | -0.04, 0.004 | 0.12 |  |
| Types of ART |  |  |  |  | |  |  |  |
| NNRTI | Ref |  |  | Ref | |  |  |  |
| PI | 0.02 | 0.003, 0.04 | 0.024 | 0.01 | | -0.01, 0.03 | 0.45 |  |
| Other | 0.03 | 0.002, 0.05 | 0.035 | -0.01 | | -0.04, 0.03 | 0.64 |  |
| exposure to stavudine | -0.01 | -0.02, 0.01 | 0.447 |  | |  |  |  |
| Fragility status |  |  |  |  | |  |  |  |
| Normal | Ref |  |  | Ref | |  |  |  |
| Pre-frailty | 0.02 | 0.01, 0.04 | 0.012 | 0.01 | | -0.01, 0.04 | 0.15 |  |
| Frailty | 0.08 | 0.05, 0.11 | <0.001 | 0.07 | | 0.04, 0.11 | **<0.001** |  |
| Diabetes mellitus was defined as fasting blood glucose ≥126 mg/dL for two consecutive visits of 6 months interval or a physician diagnosis or taking anti-diabetes medications. Hypertension was defined as SBP ≥140 mmHg or DBP ≥90 mmHg for two consecutive visits of 6 months interval or taking anti-hypertensive medications.  Abbreviations: aOR, adjusted odds ratio; BMI, body mass index; LDL, low-density lipoprotein; HDL, high-density lipoprotein; ALT, alanine aminotransferase; IL-6, interleukin-6. | | | | | | | |  |

**Table S4.** Univariate and multivariate logistic regression for high PhenoAge in all participants

| **All participants** | **Univariate** | | | **Multivariate** | | |  |
| --- | --- | --- | --- | --- | --- | --- | --- |
|  | **OR** | **95% CI** | **p-value** | **aOR** | **95% CI** | **p-value** |  |
| **Male** (vs. female) | 1.58 | 1.07, 2.34 | 0.021 | 1.05 | 0.66, 1.67 | 0.841 |  |
| **BMI** ≥25 kg/m^2^  (vs. <25 kg/m^2^) | 1.45 | 0.97, 2.17 | 0.071 | 0.90 | 0.55, 1.47 | 0.668 |  |
| **HIV status** | 1.06 | 0.68, 1.65 | 0.800 | 0.71 | 0.41, 1.22 | 0.211 |  |
| **Abnormal waist circumference** | 1.28 | 0.88, 1.87 | 0.194 |  |  |  |  |
| **Smoking** |  |  |  |  |  |  |  |
| Never/ ex-smoker | Ref |  |  |  |  |  |  |
| current | 2.33 | 1.29, 4.24 | 0.005 | 2.29 | 1.13, 4.64 | **0.022** |  |
| **Alcohol drinking** |  |  |  |  |  |  |  |
| Never/ ex-smoker | Ref |  |  |  |  |  |  |
| current | 0.95 | 0.51, 1.76 | 0.862 |  |  |  |  |
| **Diabetes mellitus** | 4.24 | 2.34, 7.68 | <0.001 | 2.83 | 1.45, 5.51 | **0.002** |  |
| **Hypertension** | 2.98 | 1.99, 4.47 | <0.001 | 2.26 | 1.41, 3.62 | **0.001** |  |
| **Statin use** | 1.30 | 0.86, 1.96 | 0.219 |  |  |  |  |
| **HDL-cholesterol**  ≤40 mg/dL (vs. >40 mg/dL) | 1.34 | 0.88, 2.04 | 0.174 |  |  |  |  |
| **LDL-cholesterol**  ≥130 mg/dL (vs. <130 mg/dL) | 0.86 | 0.59, 1.26 | 0.436 |  |  |  |  |
| **Triglycerides**  ≥150 mg/dL (vs. <150 mg/dL) | 1.03 | 0.71, 1.5 | 0.885 |  |  |  |  |
| **ALT** >50 (vs. ≤50 IU/mL) | 0.93 | 0.55, 1.56 | 0.774 |  |  |  |  |
| **Insulin**, IU/mL (per unit increase) | 1.03 | 1.01, 1.06 | 0.014 |  |  |  |  |
| **IL-6**, pg/ml (per unit increase) | 1.04 | 1.01, 1.07 | 0.008 | 1.04 | 1.01, 1.09 | **0.024** |  |
| **Frailty status** |  |  |  |  |  |  |  |
| Normal | Ref |  |  | Ref |  |  |  |
| Pre-frailty | 1.43 | 0.95, 2.18 | 0.09 | 1.44 | 0.91, 2.28 | 0.116 |  |
| Frailty | 4.78 | 1.94, 11.75 | 0.001 | 3.89 | 1.44, 10.51 | **0.007** |  |
| Diabetes mellitus was defined as fasting blood glucose ≥126 mg/dL for two consecutive visits of 6 months interval or a physician diagnosis or taking anti-diabetes medications. Hypertension was defined as SBP ≥140 mmHg or DBP ≥90 mmHg for two consecutive visits of 6 months interval or taking anti-hypertensive medications.  Abbreviations: aOR, adjusted odds ratio; BMI, body mass index; LDL, low-density lipoprotein; HDL, high-density lipoprotein; ALT, alanine aminotransferase; IL-6, interleukin-6. | | | | | | | |

**Table S5**. Comparison of phenotype age between participants with frailty and those without frailty, stratified by HIV status

| **Phenotypic age** |  |  |  |  |
| --- | --- | --- | --- | --- |
| HIV-negative | Frailty | n | Mean (SD) | Median (IQR) |
|  | Normal | 53 | 48.68 (7.20) | 48.38 (43.92, 51.43) |
|  | Pre-Frailty | 40 | 48.42 (6.86) | 45.95 (43.71, 52.29) |
|  | Frailty | 2 | 64.26 (1.19) | 64.26 (63.42, 65.1) |
|  | p-value=N/A |  |  |  |
| HIV-positive |  | n | Mean (SD) | Median (IQR) |
|  | Normal | 105 | 46.68 (7.13) | 45.20 (41.5, 50.71) |
|  | Pre-Frailty | 169 | 49.42 (8.85) | 48.72 (42.67, 54.74) |
|  | Frailty | 29 | 56.86 (10.84) | 55.51 (50.22, 62.87) |
|  | P-value*= 0.0001 |  |  |  |
| *P-value by Kruskal-Wallis test | | | | |
| **PAA** | | | | |
| HIV-negative | Frailty | n | Mean (SD) | Median (IQR) |
|  | Normal | 53 | -8.12 (5.45) | -9.22 (-11.48, -6.43) |
|  | Pre-Frailty | 42 | 1.19 (10.74) | 1.19 (-6.41, 8.78) |
|  | Frailty | 2 | -8.12 (5.45) | -9.22 (-11.48, -6.43) |
|  | p-value= N/A |  |  |  |
| HIV-positive |  | n | Mean (SD) | Median (IQR) |
|  | Normal | 105 | -7.80 (5.95) | -8.66 (-11.31, -5.23) |
|  | Pre-Frailty | 169 | -6.77 (6.72) | -7.80 (-11.18, -3.13) |
|  | Frailty | 29 | -2.66 (7.58) | -3.00 (-7.19, 0.85) |
|  | P-value*= 0.0010 |  |  |  |
| *P-value by Kruskal-Wallis test | | | | |

**Table S6**. Characteristics of PLWH and HIV-negative controls

| **Total N= 435** | **PLWH (N=333)** | **HIV-negative (N=102)** | **P-value** |
| --- | --- | --- | --- |
| **Age** | 54.4 (51.7-59.4) | 55.4 (52.9-57.8) | 0.38 |
| **Sex** |  |  | 0.94 |
| Male | 207 (62.16) | 63 (61.76) |  |
| Female | 126 (37.84) | 39 (38.24) |  |
| **Body mass index** (BMI), kg/m^2^ | 23.1(20.8-25.3) | 25.3 (22.3-28.0) | <0.001 |
| **Waist circumference**, cm | 84 (78- 90) | 87 (80-93) | 0.017 |
| **Education** |  |  | 0.12 |
| No Education | 4 (1.2) | 0 (0) |  |
| Primary/Secondary school | 125 (37.54) | 51 (50) |  |
| High School / Vocational | 101 (30.33) | 23 (22.55) |  |
| Higher than bachelor’s degree | 103 (30.93) | 28 (27.45) |  |
| **Employment** |  |  | 0.030 |
| Unemployed | 59 (17.72) | 9 (8.82) |  |
| Employed | 274 (82.28) | 93 (91.18) |  |
| **Income group (THB, monthly)** |  |  | 0.017 |
| No income | 32 (9.61) | 7 (6.86) |  |
| <10,000 | 99 (29.73) | 18 (17.65) |  |
| 10,000-19,999 | 100 (30.03) | 30 (29.41) |  |
| >=20,000 | 102 (30.63) | 47 (46.08) |  |
| **Low physical activity** |  |  | 0.054 |
| No | 201 (66.34) | 73 (76.84) |  |
| Yes | 102 (33.66) | 22 (23.16) |  |
| **Smoking** |  |  | 0.76 |
| Never | 213 (63.96) | 68 (66.67) |  |
| Ex-smoker | 75 (22.52) | 23 (22.55) |  |
| Current | 45 (13.51) | 11 (10.78) |  |
| **Alcohol drinking** |  |  | 0.21 |
| Never | 260 (78.08) | 77 (75.49) |  |
| Ex-drinker | 43 (12.91) | 10 (9.8) |  |
| Current | 30 (9.01) | 15 (14.71) |  |
| **Illicit drug use** |  |  |  |
| Ever use | 6/321 (1.87) | 0 /99 (0) | 0.34 |
| Current use | 3/321 (0.93) | 0 /99 (0) | 1.00 |
| **HBV infection** | 42 (12.61) | 6 (5.88) | 0.070 |
| **HCV infection** | 30 (9.01) | 3 (2.94) | 0.052 |
| **Diabetes mellitus** | 55 (16.52) | 16 (15.69) | 0.84 |
| **Hypertension** | 141 (42.34) | 26 (25.49) | 0.002 |
| **Cardiovascular diseases (CVD)** | 17 (5.11) | 1 (0.98) | 0.087 |
| **Current statin use** | 109 (32.73) | 19 (18.63) | 0.006 |
| **Total cholesterol**, mg/dL | 203 (180-238) | 220 (196-245) | 0.003 |
| **HDL-cholesterol**, mg/dL | 46 (39-57) | 51 (45-60) | 0.0004 |
| **LDL-cholesterol**, mg/dL | 123 (100-148) | 141 (116 -162) | 0.0001 |
| **Triglycerides**, mg/dL | 155 (104-219) | 121 (93-168) | 0.0001 |
| **Creatinine**, mg/dL | 0.87 (0.76-1.04) | 0.84 (0.73-0.95) | 0.026 |
| **eGFR (CKD-EPI)**, mL/min per 1.73m2 | 88.58 (74.01-98.28) | 93.76 (85.63-97.83) | 0.012 |
| **ALT**, IU/mL | 28 (21-39) | 25 (18-36) | 0.031 |
| **AST**, IU/mL | 25 (21-32) | 24 (19-28) | 0.009 |
| **ALP**, IU/mL | 3.4 (3-3.8) | 3.2 (2.8-3.4) | <0.001 |
| **APRI**, IU/mL | 0.25 (0.19-0.33) | 0.21 (0.17-0.28) | 0.001 |
| **FIB-4 score** | 1.03 (0.85-1.39) | 0.93 (0.77-1.17) | 0.003 |
| **Albumin** | 44 (40-46) | 40 (38-42) | <0.001 |
| **Insulin**, IU/mL | 6.9 (4.8-10.9) | 5.2 (3.7-7.4) | <0.001 |
| **hs-CRP**, mg/dL | 0.12 (0.06-0.25) | 0.13 (0.05-0.28) | 0.83 |
| **IL-6**, pg/mL | 6.00 (4.14-7.93) | 6.31 (4.00-12.17) | 0.23 |
| **Montreal Cognitive Assessment (MOCA)** | 24 (21-26) | 23 (20-26) | 0.27 |
| Abnormal MOCA (≤24) | 201 (60.54) | 64 (62.75) | 0.69 |
| **Frailty status** |  |  | <0.001 |
| Robust | 105 (34.65) | 53 (55.79) |  |
| Pre-frail | 169 (55.78) | 40 (42.11) |  |
| Frail | 29 (9.57) | 2 (2.11) |  |

**Figure S1.** Correlation between chronological age and phenotypic age for overall population (left) and stratified by HIV status (right)


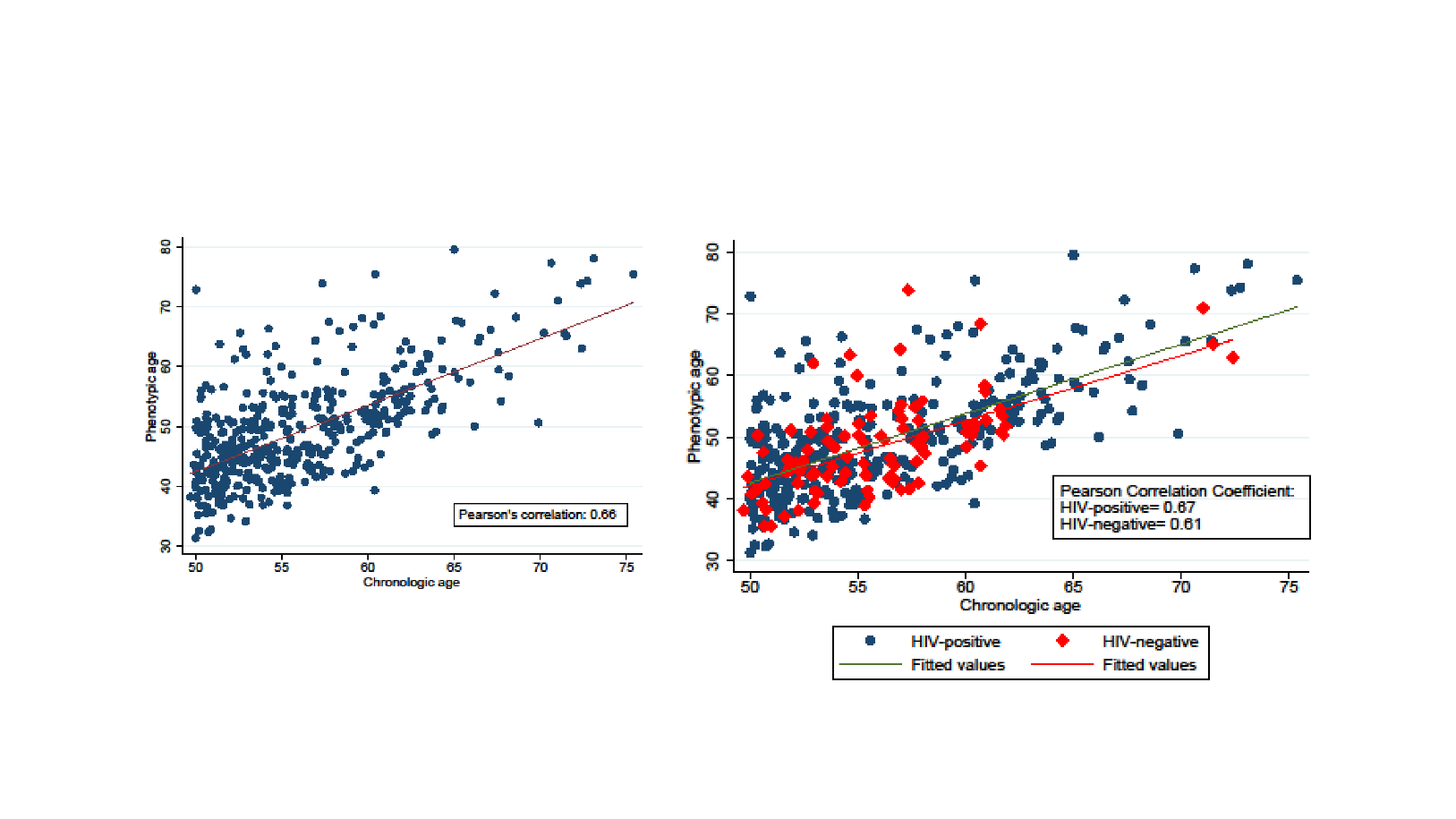


**Figure S2**. Comparison of phenotypic age (2A), chronological age (2B) and phenotypic age acceleration (2C) by HIV status and VACS index by PAA status (2D)


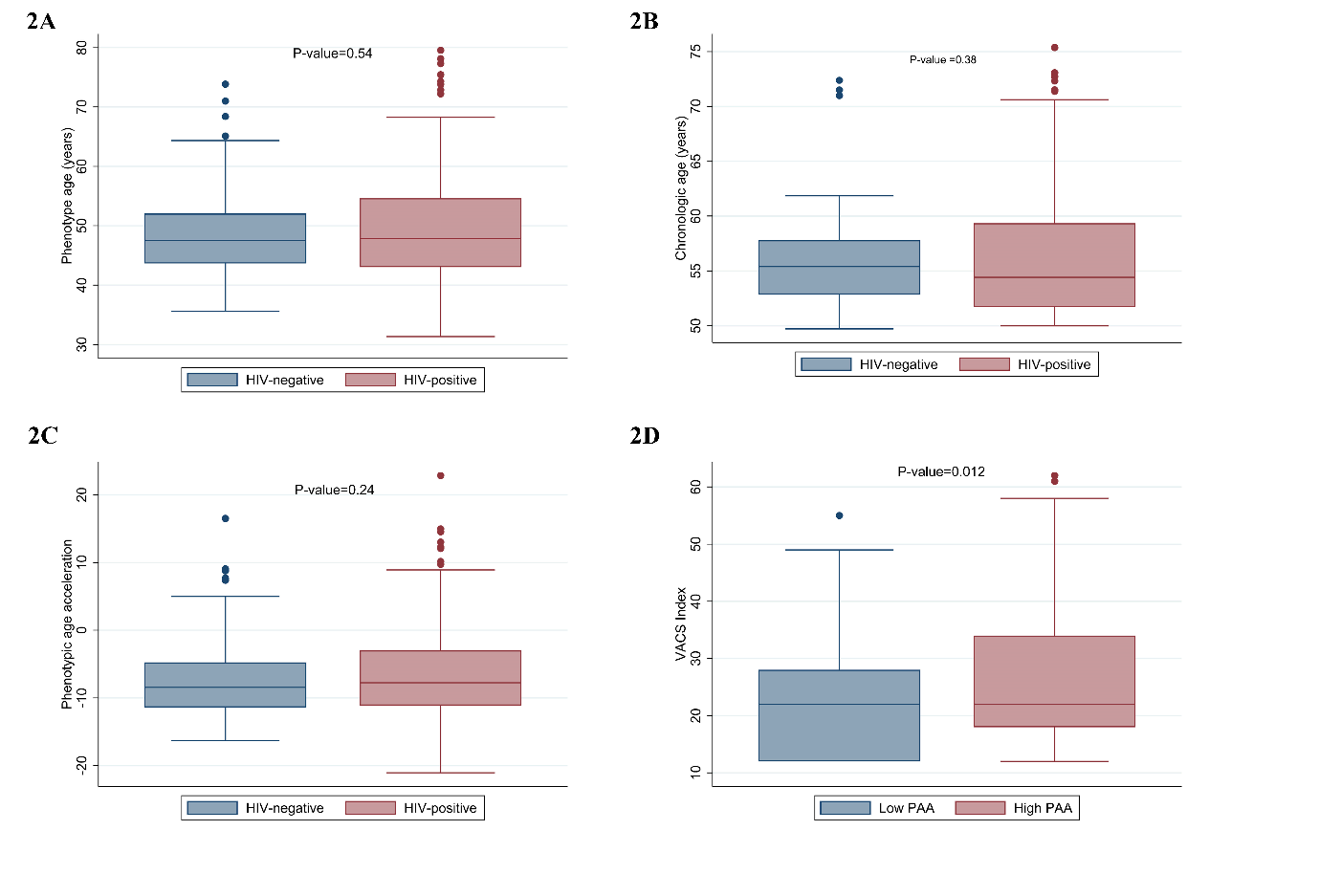


**Figure S3**. Comparison of PhenoAge and PAA between different disease counts by HIV status


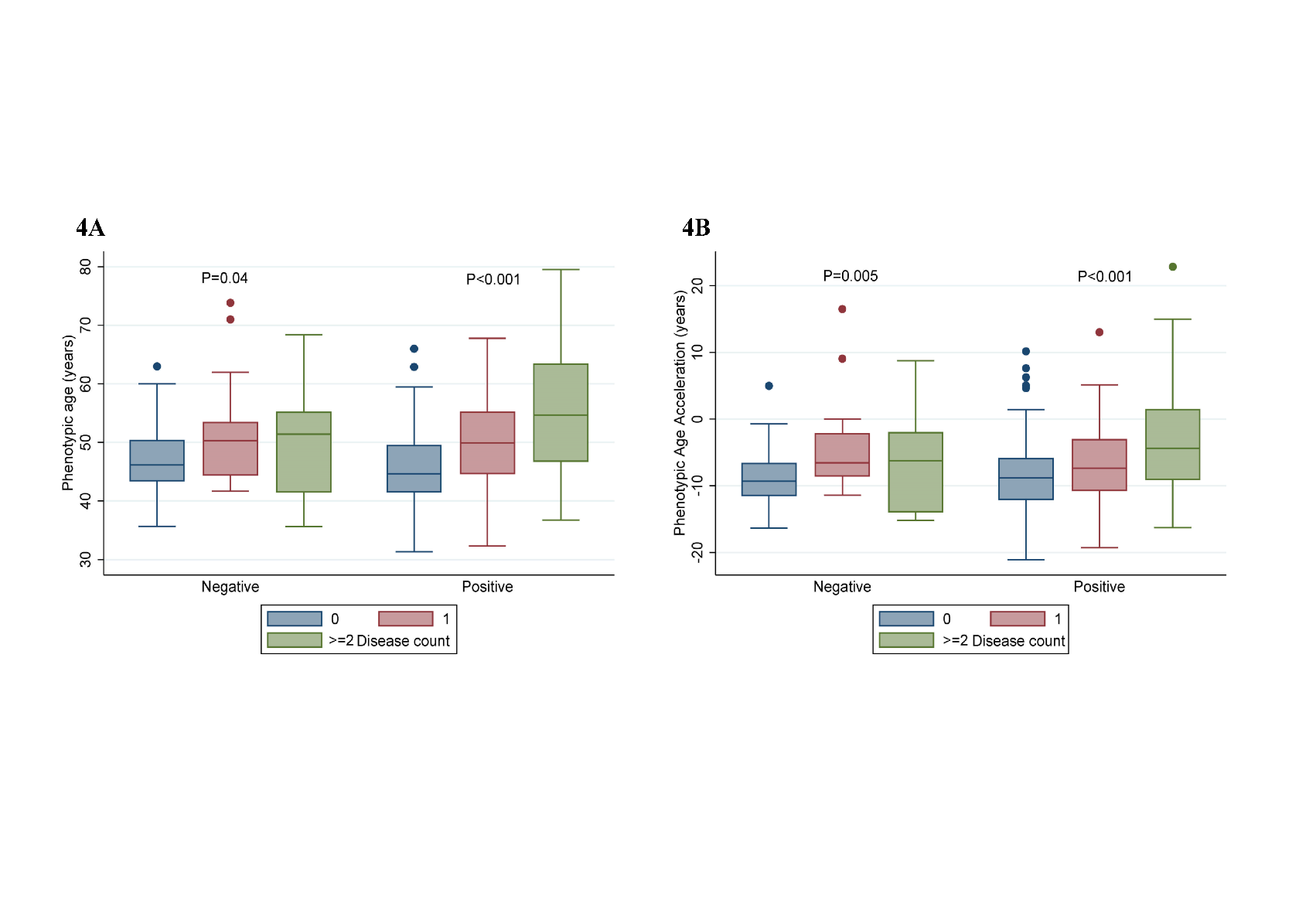


The diseases included in the disease count were hypertension, diabetes mellitus, cardiovascular diseases, frailty, chronic kidney disease (defined as estimated glomerular filtration rate <60 mL/min using CKD-EPI equation).
